# Infant Formulas With Partially or Extensively Hydrolyzed Milk Proteins for the Prevention of Allergic Diseases: A Systematic Review and Meta-Analysis of Clinical Trials

**DOI:** 10.1016/j.advnut.2024.100217

**Published:** 2024-04-04

**Authors:** Xiaoxu Li, Tingchao He, Sufang Duan, Jinghong Liang, Gang Feng, Fang Li, Zhenyu Shen, Wenhui Ye, Biao Liu, Bibo Jiang, Yujing Chen, Nan Liu, Ignatius Man-Yau Szeto, Li Cai

**Affiliations:** 1 Department of Maternal and Child Health, School of Public Health, Sun Yat-sen University, Guangzhou, Guangdong Province, China; 2 Inner Mongolia Dairy Technology Research Institute, Hohhot, China; 3 Inner Mongolia Yili Industrial Group, Yili Maternal and Infant Nutrition Institute (YMINI), Beijing, China; 4 National Center of Technology Innovation for Dairy, Hohhot, China; 5 Inner Mongolia Yili Industrial Group, Hohhot, China; 6 The First Affiliated Hospital, Sun Yat-sen University, Guangzhou, Guangdong Province, China; 7 Guangdong Provincial Key Laboratory of Food, Nutrition and Health, School of Public Health, Sun Yat-sen University, Guangzhou, China

**Keywords:** allergy, prevention, partially hydrolyzed milk protein formula, extensively hydrolyzed milk protein formula, meta-analysis

## Abstract

Despite the widely recommended usage of partially hydrolyzed formula (PHF) or extensively hydrolyzed formula (EHF) of milk protein for preventing allergic diseases (ADs), clinical studies have been inconclusive regarding their efficacy compared with that of cow's milk formula (CMF) or breast milk (BM). We aimed to systematically evaluate the effects of PHF or EHF compared with those of CMF or BM on risk of ADs (cow's milk allergy, allergic rhinitis, eczema, asthma, wheeze, food allergy, and sensitization) in children. We searched PubMed, Embase, Cochrane Library, and Web of Science for clinical trials published from inception to 21 October, 2022. We used the Grading of Recommendations Assessment, Development, and Evaluation (GRADE) approach to grade the strength of evidence. Overall, 24 trials (10,950 infants) were included, 17 of which specifically included high-risk infants. GRADE was low for the evidence that, compared with CMF, infants early fed with EHF had lower risk of cow's milk allergy at age 0–2 y [relative risk (RR): 0.62; 95% CI: 0.39, 0.99]. Moderate evidence supported that PHF and EHF reduced risk of eczema in children aged younger or older than 2 y, respectively (RR: 0.71; 95% CI: 0.52, 0.96; and RR: 0.79; 95% CI: 0.67, 0.94, respectively). We also identified moderate systematic evidence indicating that PHF reduced risk of wheeze at age 0–2 y compared with CMF (RR: 0.50; 95% CI: 0.29, 0.85), but PHF and EHF increased the risk compared with BM (RR: 1.61; 95% CI: 1.11, 2.31; and RR: 1.64; 95% CI: 1.26, 2.14). Neither PHF nor EHF had significant effects on other ADs in children of any age. In conclusion, compared with CMF, PHF, or EHF had different preventive effect on cow's milk allergy, eczema, and wheeze. Compared with BM, both PHF and EHF may increase risk of wheeze but not other ADs. Given that most trials included only high-risk infants, more research on non–high-risk infants is warranted before any generalization is attempted.

This protocol was registered at PROSPERO as CRD42022320787.

**Statement of Significance**

To our knowledge, this meta-analysis is the first to comprehensively compare the preventive effect of partially or extensively hydrolyzed milk protein formula with cow's milk formula or breast milk on risk of allergic diseases.

## Introduction

Allergic diseases (ADs) represent a global public health concern owing to the persistent health-threatening and socioeconomic burden [1]. ADs often first occur in childhood, and their prevalence has been rising in recent years [2]. Besides genes [3], environmental factors such as external toxins and allergens, microbiome, as well as early nutrition affect children’s risk of developing ADs [4,5]. Among them, newborn feeding practices have a significant impact on early childhood nutrition [6]. Infant feeding is considered the first modifiable factor in early life and an important target for individualized interventions for childhood diseases [7]. Therefore, early infant feeding is crucial for achieving primary prevention of ADs.

Cow's milk formula (CMF) for infants is commonly used as a substitute for breast milk (BM) when breastfeeding is not possible or insufficient [8]. However, for infants with cow's milk allergy, hydrolyzed milk formula (HF) is recommended as the primary option for its treatment and management [9,10]. Compared with CMF, HF contains tiny peptides that are easier to digest, thereby lessening sensitivity and reducing the likelihood of allergic reactions. Partially hydrolyzed formula (PHF) and extensively hydrolyzed formula (EHF) are 2 types of HFs that vary in the extent of protein hydrolysis [11]. Previous clinical trials have examined the effect of PHF and EHF on risk of ADs and yielded mixed results. There are some studies [12,13] showing a reduction in risk of eczema in high-risk infants fed HF, whereas others show no significant effect [14,15].

Previous meta-analyses of clinical trials have suggested that EHF compared with CMF may prevent infant cow's milk allergy and eczema. However, these studies were limited by several factors, such as the unequal distribution of interventions among groups [16], the focus on a single kind of HF or AD [17], and the inclusion of a small number of studies [18]. As such, the effect of early feeding with HF compared with that of CMF on the development of ADs remains inconclusive. On the contrary, although HF can serve as a supplement or substitute for BM, meta-analysis comparing the effects of HF and BM on risk of ADs in infants and children is currently lacking.

Therefore, this study conducted a comprehensive meta-analysis of clinical trials to investigate the effects of PHF or EHF compared with those of CMF or BM in infants and children. The prevalence of ADs was dependent on age and varied by the type of allergic disease. Therefore, all meta-analyses were conducted for different types of ADs in children aged younger or older than 2 y, according to the time points of outcomes reported in the original study.

## Methods

### Search strategy and selection criteria

The study followed the PRISMA guidelines [19] for reporting meta-analysis. This protocol was registered at PROSPERO (CRD42022320787).

We searched 4 electronic databases (PubMed, Embase, Web of Science, and Cochrane Library) for relevant studies published from inception to 21 October, 2022. The detailed search strategy is provided in Supplemental Table 1. We also manually searched the reference lists of the included studies and relevant systematic reviews. Only English-language articles were included.

The study adhered to the population, interventions, comparators, outcomes, study design criteria for the included studies. First, only infants without any clinical diagnosis of ADs were recruited. Second, the intervention groups were given PHF or EHF or their subtypes, including partially hydrolyzed whey (PHF-W) or partially hydrolyzed casein (PHF-C) formulas and extensively hydrolyzed whey (EHF-W) or extensively hydrolyzed casein (EHF-C) formulas. Third, the control groups were provided with either CMF or BM. Fourth, the outcomes included cow's milk allergy, atopic dermatitis/eczema, allergic rhinitis, asthma, wheeze, food allergy, or sensitization. Finally, the included studies were randomized controlled trials (RCTs) and quasi-RCTs, as well as controlled clinical trials.

We excluded therapeutic trials targeting only infants with ADs at baseline, as well as preventive trials with outcomes unrelated to ADs. In addition, studies that solely applied multiple allergy prevention measures, such as maternal diet restriction and avoidance of aeroallergens, to the intervention group were excluded. However, studies that implemented allergy prevention measures, such as encouraging breastfeeding or delaying the introduction of solid foods, in both the intervention and control groups were eligible for inclusion.

### Data extraction and assessment of risk of bias

Two authors (XXL, JHL) independently screened the titles and abstracts and reviewed the full-text articles to determine eligibility for inclusion. One author used a standardized data collection form to extract information and outcome data from the included studies, whereas the other author verified the completeness and accuracy of the extracted data. Any inconsistencies or uncertainties were resolved through a team meeting to reach a consensus.

Two authors (XXL, JHL) evaluated the quality of eligible studies using the Cochrane Risk of Bias (ROB) tool [20], which covered 7 items: random sequence generation, allocation concealment, blinding of participants and personnel, blinding of outcome assessment, incomplete outcome data, selective reporting, and other biases. Each item from the included studies was then graded as having uncertain, low, or high ROB.

### Data selection for analysis

First, to minimize reporting bias, we included data from only 1 article if multiple articles were from the same trial. In our included 24 trials, 4 trials published 1 or more articles [[21], [22], [23], [24], [25], [26], [27], [28], [29], [30], [31]]. The German Infant Nutritional Intervention (GINI) trails yielded 5 articles [[21], [22], [23], [24], [25]], and we used data only from the 15-y follow-up article [25] because it provided the most comprehensive information on allergic outcomes. The remaining 3 trials each published 2 articles [[26], [27], [28], [29], [30], [31]]. We selected articles reporting clinical diagnosis outcomes (e.g., cow's milk allergy) [26,28,30] over those focusing on serologic assessments (e.g., S-IgE). The details of these studies are provided in Supplemental Table 2.

Second, to ensure data accuracy and avoid bias, we followed a systematic approach when an article reported multiple follow-up time points within our predetermined age groups. We selected the data that had the most complete information, largest number of events, or most observed outcomes. In addition, in cases where a study had multiple intervention groups, we performed pairwise comparisons and divided the number of events and non-events to prevent duplicate data.

### Data synthesis and meta-analysis

We performed meta-analyses using random-effect models to calculate the relative risk (RR) with 95% CIs for the effects of PHF or EHF compared with those of CMF or BM on ADs when the quantity of articles is >2. Heterogeneity among studies was quantified using the *I*^2^ statistic. All meta-analyses were conducted for different types of ADs in children aged younger or older than 2 y, according to the time points of outcomes reported in the included studies. Subgroup analyses with 6 or more articles were performed, differentiating between high-risk infants and non–high-risk infants, as well as between casein-dominant and whey-dominant hydrolysates. High-risk infants were defined as those with a first-degree relative having a history of ADs at birth [32]. We performed several sensitivity analyses to confirm our findings: *1*) investigate the influence of individual studies on the pooled effects; *2*) replace articles from different follow-up periods of the GINI trial to re–meta-analysis; and *3*) exclude articles involving preterm or low birth weight infants to re–meta-analysis. The publication bias was assessed using the Egger test and Begg test and funnel plot when the quantity of articles is >10. We considered a *P* value of <0.05 for statistical significance. Review Manager version 5.3 and STATA version 12 were used for this meta-analysis.

### Strength of the body of evidence

We applied the Grading of Recommendations Assessment, Development and Evaluation [33] (GRADE) approach to evaluate the quality of evidence. The evidence from RCTs was deemed to be of high quality. However, we downgraded the evidence by 1 level for serious limitations and by 2 levels for very serious limitations based on criteria, including the ROB in the study design, consistency across studies, directness of evidence, precision of estimates, and potential publication bias. Finally, the evidence was graded into 1 of the following 4 levels: *1*) high quality: further research is very unlikely to change our confidence in the estimate of effect; *2*) moderate quality: further research is likely to have an important impact on our confidence in the estimate of effect and may change the estimate; *3*) low quality: further research is very likely to have an important impact on our confidence in the estimate of effect and is likely to change the estimate; and *4*) very low quality: we are very uncertain about the estimate.

## Results

### Literature flow and study characteristics

**FIGURE 1:**
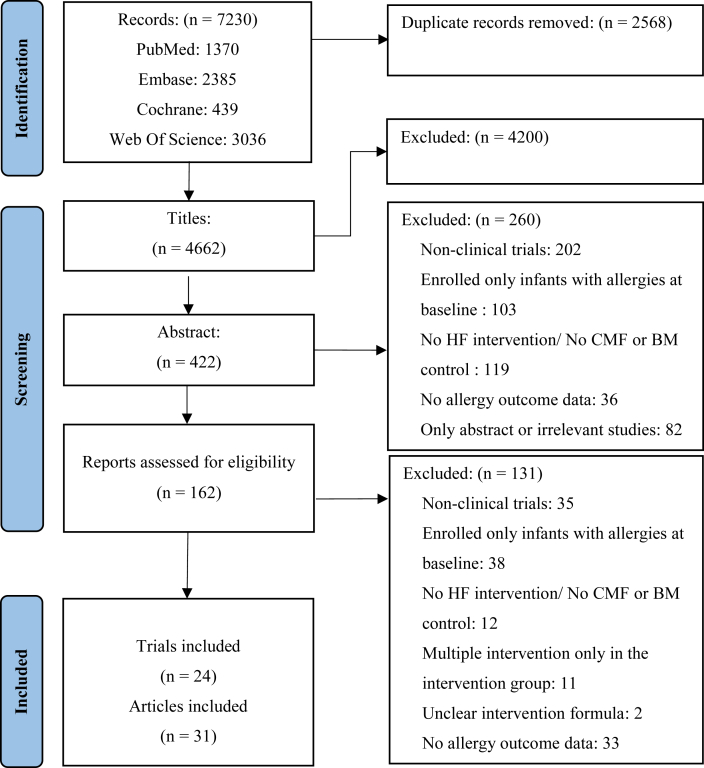
Flow chart of the study selection for the meta-analysis. Flow chart of the study selection for the meta-analysis, and main reasons for exclusions. After screening titles, abstracts, and full-text articles, 24 studies met the inclusion criteria for meta-analysis. BM, breast milk; CMF, cow's milk formula; HF, hydrolyzed formula.

A total of 24 studies [[13], [14], [25], [26], [28], [30], [34], [35], [36], [37], [38], [39], [40], [41], [42], [43], [44], [45], [46], [47], [48], [49], [50], [51]] met the inclusion criteria for meta-analysis after screening 7230 records (Figure 1). Table 1 summarizes the study characteristics of included studies. These clinical trials involved 10,950 infants, comprising 17 RCTs [13,14,25,28,[34], [35], [36], [37], [38],[41], [42], [43],[45], [46], [47], [48],^51^], 6 quasi-RCTs [26,30,39,40,44,50], and 1 controlled clinical trial [49].The sample size ranged from 33 to 4284, with a mean of 456 infants per trial. Among these studies, 10 studies used only PHF in early infancy [13,28,35,38,39,42,46,47,49,51], 8 studies used only EHF [14,26,30,34,36,[43], [44], [45]], and 6 studies used both PHF and EHF [25,37,38,40,41,48]. In the studies using PHF, 4 studies did not provide information about the type of hydrolyzed protein [13,[39], [40], [41]]. Among the remaining 12 studies [25,28,35,37,38,42,[46], [47], [48], [49], [50], [51]], all used PHF-W as intervention, and none used PHF-C. For studies using EHF, 4 did not specify the type of hydrolyzed protein [34,37,40,41] and 5 used only EHF-C [14,26,36,45,48], 2 used only EHF-W [30,43], and 3 used both EHF-C and EHF-W [25,44,50]. Seventeen trials specifically involved infants at high risk [13,25,28,35,[37], [38], [39], [40], [41], [42], [43], [44], [45], [46], [47], [48],^50^], whereas 1 trial included non–high-risk and high-risk infants [51]. The intervention duration ranged from 0 to 8 mo, and the outcomes were ascertained from 1 mo to 15 y of age. Common ADs reported in the 24 studies included cow's milk allergy (25%), allergic rhinitis (25%), eczema/allergic dermatitis (75%), asthma (33.3%), wheeze (29.2%), food allergy (16.7%), and sensitization (45.8%).

**TABLE 1:** Study characteristics of the included studies

ID	Study	Design	Country	Population	Participant (*n*)	Formula type	Intervention duration	Age at outcome	Outcome measures^1^	Allergic outcomes	Main findings
1	Nicolaou et al. [51] 2022	RCT	Bulgaria; Cyprus; Greece	High-risk and non–high-risk term infants	331	PHF-W/CMF	First 6 mo	6 mo	Physical examination; SCORAD; and CoMiSS	CMA and eczema	AD incidence was significantly lower in those receiving PHF than in those receiving CMF (RR: 0.54; 95% CI: 0.32, 0.92)
2	Lowe et al. [35], 2011	RCT	Australia	High-risk term infants	412	PHF-W/CMF	At 6 mo of life	2 y; 6–7 y	Parent report	CMA; eczema; allergic rhinitis; asthma; and sensitization	There was no evidence that infants allocated to the PHF-W (OR: 1.21; 95% CI: 0.81, 1.80) were at lower risk of allergy in infancy than those to CMF
3	Chan et al. [13], 2002	RCT	Singapore	High-risk term infants	153	PHF/CMF	First 4 mo of life	4 mo; 6 mo; 1 y; 1.5; 2 y; and 2.5 y	Parent report; pediatricians diagnosis	Eczema; wheeze; and allergic rhinitis	The cumulative incidence and prevalence of atopic dermatitis at the age of 6 mo were significantly less in the PHF group than those in the CMF group (*P* < 0.05)
4^2^	Vandenplas et al. [28], 1995	RCT	Belgium	High-risk term infants	58	PHF-W/CMF	First 6 mo	6 mo; 1 y; 3 y; and 5 y	Parent report; skin prick test	Eczema; wheeze; allergic rhinitis; and sensitization	At 6 mo, the prevalence of cow milk protein sensitivity decreased significantly in the hydrolysate group (*P* = 0.002); at 12 mo, *P* = 0.029; 36 mo, *P* = 0.018; and 60 mo, *P* = 0.016 There was still a significant difference in the number of atopic manifestations
5	Tsai et al. [46], 1991	RCT	Taiwan	High-risk term infants	33	PHF-W/CMF	From 1–2 to 6 mo	1 y	Pediatrician diagnosis	Eczema; wheeze; and allergic rhinitis	High-risk newborns fed hypoallergenic milk showed lower incidence of allergic diseases (eczema and rhinitis) There was no different in the incidence of wheeze in the 2 groups
6	Willemset al. [49], 1993	CCT	Belgium	Non–high-risk term infants	122	PHF-W/CMF	3 mo	3 mo; 1 y	IgE; RAST	Sensitization	The investigators recommend these hypoallergenic milks (PHF) for prevention against atopy in infants at risk
7^2^	von Berg et al. [25], 2016	RCT	Germany	High-risk term infants	2252	PHF-W/EHF-W /EHF-C/CMF	First 6 mo of life	15 y	ISAAC; physical examination	Allergicrhinitis; asthma; eczema; food allergy; and sensitization	The prevalence of asthma reduced in the EHF-C group compared with that in the CMF group (OR: 0.49; 95% CI: 0.26, 0.89) The cumulative incidence of AR was lower in EHF-C (RR: 0.77; 95% CI: 0.59, 0.99) and the AR prevalence in PHF-W (OR: 0.67; 95% CI: 0.47, 0.95) and EHF-C (OR: 0.59; 95% CI: 0.41, 0.84) The cumulative incidence of eczema reduced in PHF-W (RR: 0.75; 95% CI: 0.59, 0.96) and EHF-C (RR: 0.60; 95% CI: 0.46, 0.77)
8	Virtanen et al. [14], 2021	RCT	Sweden; Canada	Non–high-risk term infants	1106	EHF-C/CMF	First 6–8 mo of life	9–11 y	ISAAC	Allergicrhinitis; eczema; and asthma	Risk of asthma, allergic rhinitis, or atopic eczema did not differ by treatment (HR: 1.00; 95% CI: 0.66, 1.52; HR: 0.95; 95% CI: 0.66, 1.38; and HR: 0.89; 95%: 0.70, 1.15, respectively)
9	Di Mauro et al. [34], 2020	RCT	Italy	Non–high-risk preterm infants	60	EHF/CMF	2 wk	3 y	Parental questionnaires; skin prick test	Eczema; asthma; and food allergy	No group differences in the incidence of atopic dermatitis, asthma, and food sensitization were found
10	Kwinta et al. [36], 2009	RCT	Poland	Non–high-risk VLBW infants	74	EHF-C/CMF	First month of life	5–7 y	ISAAC; pediatrician diagnosis	Wheeze and sensitization	Prevalence of obvious allergic diseases was not significantly different between the studied groups (RR: 1.76; 95% CI: 0.76, 4.09)
11	Mallet and Henocq [45], 1992	RCT	France	High-risk term infants	177	EHF-C/CMF	First 4 mo of life	4 mo; 1 y; 2 y; and 4 y	IgE; RAST	CMA; eczema; and asthma	At 4 y of age, allergic signs were found in 11 children in the hydrolysate group and in 17 children in the CG; the difference was significant only for eczema (*P* < 0.01)
12	Han et al. [39], 2003	q-RCT	Korea	High-risk term infants	69	PHF/CMF/BM	First 6 mo of life	6 mo	SASSAD	Eczema	The cumulative incidence and prevalence of atopic dermatitis at the age of 6 mo were significantly less in the PHF group than those in the CMF group (47% vs. 78%; *P* < 0.05; 20% vs. 59%; *P* < 0.05). The rates of the PHF group were also less than those of the BM group, but they were not statistically significant
13	Oldaeus et al. [41], 1997	RCT	Sweden	High-risk term infants	176	EHF/PHF/CMF/BM	Weaning period to 9 mo	9 mo and 1.5 y	Parents report	Eczema; wheeze; asthma; food allergy; and sensitization	From 6 to 18 mo, there were significantly less cumulative atopic symptoms in the EHF group compared with the those in the CMF group and significantly less than the PHF group until 9 mo (EHF = 34%, PHF = 58%)
14	Marini et al. [42], 1996	RCT	Italy	High-risk term infants	219	PHF/CMF/BM	First 5 mo	1 y; 2 y; and 3 y	Clinical diagnosis	Eczema; wheeze; and allergic rhinitis	All preventive measures used in this study (exclusive breastfeeding and/or hydrolyzed milk feeding) were effective at the third year of follow-up, greatly reducing allergic manifestations in high atopic risk babies in comparison with those not receiving these interventions
15	Chandra[38], 1997	RCT	Canada	High-risk term infants	216	PHF-W/CMF/BM	First 6 mo of life	0–5 y	Clinical diagnosis	Allergicrhinitis; asthma; and sensitization	Follow-up until 5 y of age showed a significant lowering in the cumulative incidence of atopic disease in the PHF group (OR: 0.322; 95% CI: 0.159, 0.653) compared with that in the CMF group
16	Chirico et al. [47], 1997	RCT	Italy	High-risk term infants	51	PHF-W/CMF/BM	First 6 mo of life	6 mo	IgE; RAST	Eczema	PHF was less antimitogenic and antigenic than CMF and was as immunogenic and antigenic asBM
17	Szajewska et al. [37], 2004	RCT	Poland	High-risk preterm infants	122	EHF/PHF/CMF/BM	4–5 mo	4–5 mo and 1 y	Pediatrician diagnosis	Eczema; wheeze; and sensitization	Use of the extensively hydrolyzed compared with a standard preterm formula significantly reduced the incidence of atopic dermatitis observed at 12mo
18^2^	Saarinen et al. [30], 2000	q-RCT	Finland	Non–high-risk term infants	6205	EHF-W/CMF/BM	First 2 mo	2 mo and 6–7 mo	Cow milk elimination-challenge test	CMA and Sensitization	The cumulative incidence of CMA in the CMF group was 2.4% compared with 1.7% in the pasteurized human milk group (OR: 0.70; 95% CI: 0.44, 1.12) and 1.5% in the whey hydrolysate group (OR: 0.61; 95% CI: 0.38, 1.00)
19^2^	Juvonen et al. [26], 1996	q-RCT	Sweden	Non–high-risk term infants	144	EHF-C/CMF/BM	First 3 d	3 y	Parents report; skin prick test	CMA; eczema; asthma; and sensitization	No differences were found in CMA between the 3 groups
20	Odelram et al. [43], 1996	RCT	Finland; Sweden	High-risk term infants	91	EHF-W/CMF/BM	6-12 mo	1.5 y	Questionnaires; physical examinations; skin prick tests, and IgE	CMA; eczema; and sensitization	The frequency of allergic/atopic disease was similar in the3 groups. However, all 3 infants who developed CMA with skin symptoms belonged to the CMF group
21	Nentwich et al. [40], 2001	q-RCT	Czech Republic	High-risk term infants	69	PHF/EHF/BM	PHF: 127.9d; EHF: 111.1 d	6 mo	Parent report	Eczema	A significantly decreased proliferation to cow milk caseins was found in the PHF group compared with that in the exclusively breastfed group
22	Porch et al. [48], 1998	RCT	New Orleans	High-risk term infants	126	EHF-C/PHF-W/ BM	First year of life	0–1 y	Clinical diagnosis	Eczema and food allergy	Without significant differences in the number of children with formula changes and positive challenges across all feeding groups
23	Halken et al. [44], 1993	q-RCT	Denmark	High-risk term infants	141	EHF-C/EHF-W/BM	First 6mo EHF-C: 4.5 wk; EHF-W: 5.8 wk	1.5 y	Clinical diagnosis	CMA; eczema; wheeze; and sensitization	The incidence of CMA the group fed EHF was 3.6%, which was a significant reduction compared with 20% in an identically defined high-risk group without dietary preventive measures (BM group)
24	Halken et al. [50], 2000	q-RCT	Denmark	High-risk term infants	478	EHF-C/EHF-W/PHF-W/BM	At the first 4 mo of life	1.5 y	Pediatrician diagnosis	CMA; asthma; allergic rhinitis; eczema; wheeze; food allergy; and sensitization	The cumulative incidence of confirmed CMA was 1.3% in infants fed BM, 0.6% in infants fed EHF, and 4.7% in infants fed PHF PHF was found to be less effective than EHF in preventing CMA (*P* = 0.05)

Abbreviations: BM, breast milk; CG, control group; CMA, cow milk allergy; CMF, cow milk formula; CoMiSS, comprehensive milk allergy symptom score; EHF, extensively hydrolyzed formula; EHF-C, extensively hydrolyzed casein formula; EHF-W, extensively hydrolyzed whey formula; HF, hydrolyzed formula; ISAAC, International Study of Asthma and Allergic in Children; OR, odds ratio; PHF, partially hydrolyzed formula; PHF-W, partially hydrolyzed whey formula; q-RCT, quasirandomized controlled trial; RAST, rapid annotations using subsystems technology; RCT, randomized controlled trial; RR, relative risk; SASSAD, 6-area, 6-sign atopic dermatitis; SCORAD, scoring atopic dermatitis; VLBW, very low birth weight.

1 SCORAD, CoMiSS, and SASSAD are scoring systems used to assess the severity and symptoms of eczema. ISAAC is a standardized questionnaire for evaluating childhood wheezing and asthma. RAST, IgE test, and CM elimination-challenge test are serologic testing methods for detecting allergens.

2 Trials that had published >1 article.

### HF feeding compared with CMF feeding

Twenty studies [13,14,25,26,28,30,[34], [35], [36], [37], [38], [39],[41], [42], [43],[45], [46], [47],^49^,^51^] reported the effect of HF compared with CMF on ADs. In 44 results focusing on the effect of HF on total ADs in children aged <2 y, 31 (70%) showed a favorable effect, and 5 of them (11%) were statistically significant. Among the 44 results for children aged >2 y, 29 (66%) reported an advantageous effect, where 5 (11%) were statistically significant (Supplemental Table 3). The following were the results of the meta-analysis by ADs:

#### PHF compared with CMF on allergic outcomes in children aged <2 y

**FIGURE 2:**
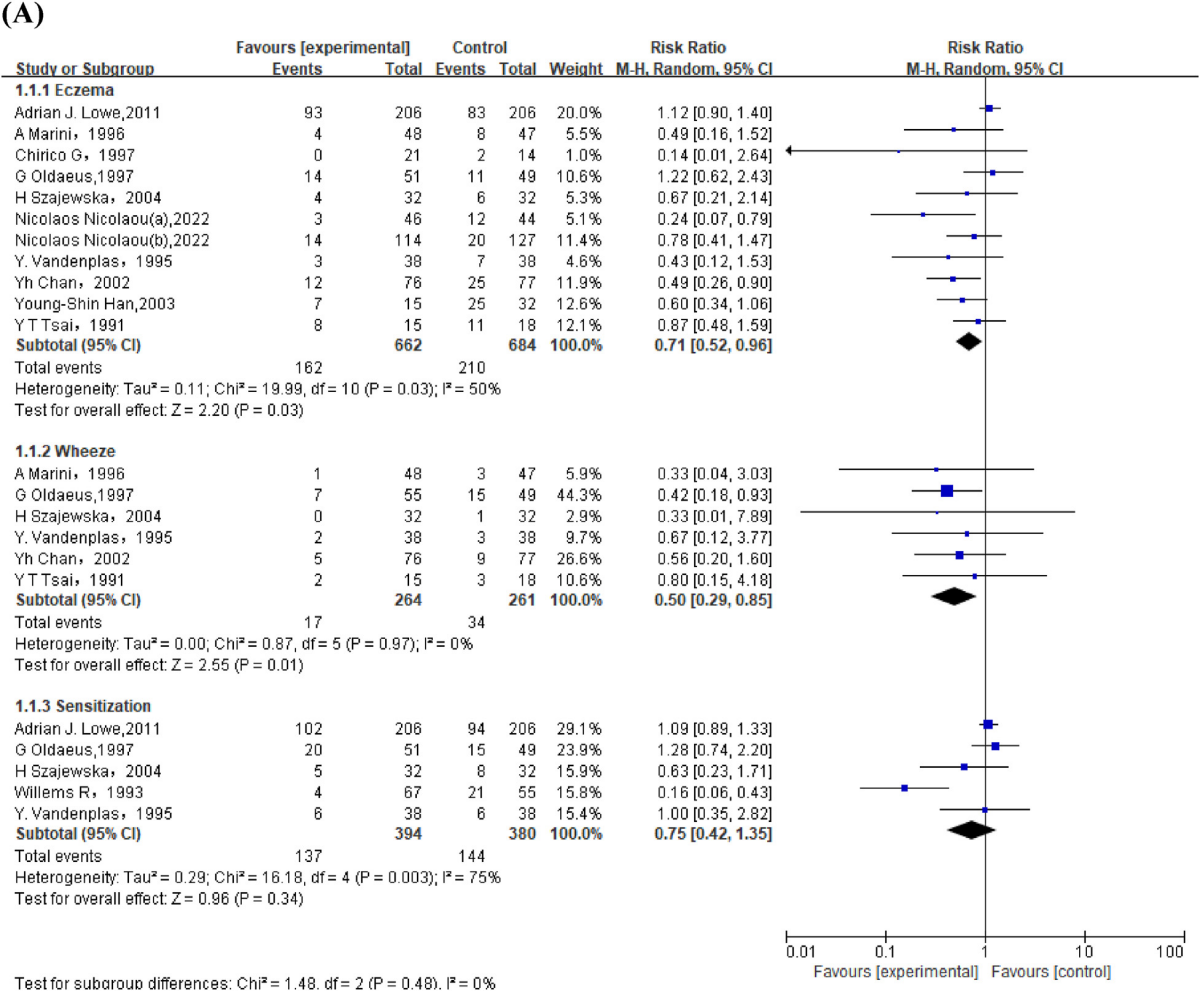

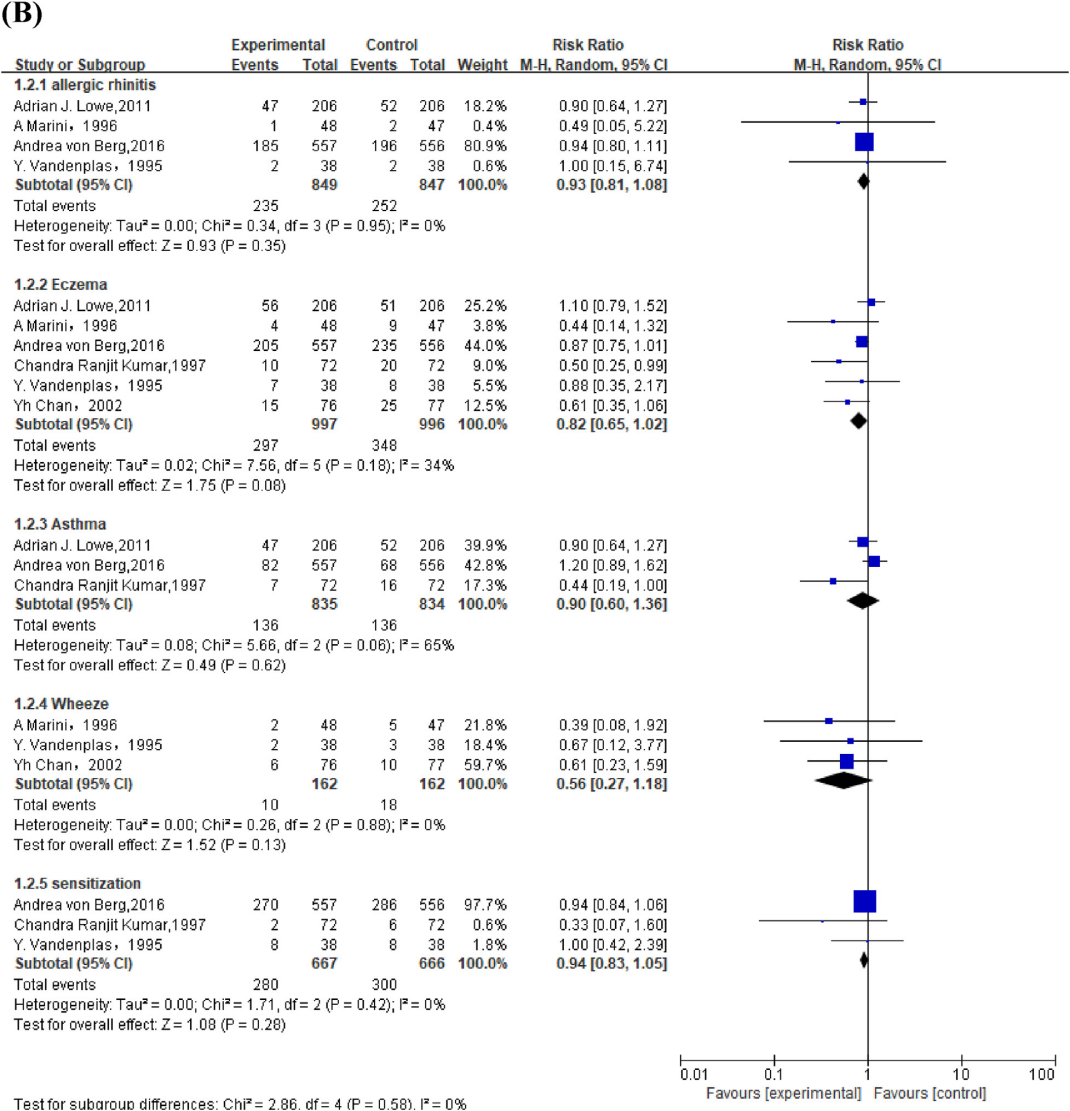
Forest plot of partially hydrolyzed formula (PHF) compared with cow's milk formula (CMF) on allergic outcomes in (A) children aged <2 y and (B) children aged >2 y. ^a^Nicolaou et al. [51] included both high-risk [Nicolaos Nicolaou (a), 2022] and non–high-risk [Nicolaos Nicolaou (b), 2022] infants. ^b^When replacing 15-y follow-up article with 3-y follow-up article in the GINI trial, sensitivity analysis found that compared with CMF, PHF reduced risk of eczema in children aged >2 y (risk ratio: 0.72; 95% CI: 0.53, 0.98).

Meta-analysis of 10 studies revealed that PHF reduced risk of eczema compared with CMF in children aged <2 y (RR: 0.71; 95% CI: 0.52, 0.96) (Figure 2A). Subgroup analysis among high-risk infants also revealed similar results (RR: 0.69; 95% CI: 0.49, 0.97) (Supplemental Figure 1). Meta-analysis of 6 studies involving 525 high-risk infants reported that PHF decreased risk of wheeze (RR: 0.50; 95% CI: 0.29, 0.85) (Figure 2A).

Overall, GRADE indicated moderate quality evidence for PHF compared with that of CMF on eczema because 9 of the 10 included articles demonstrated low or unclear selection and reporting bias. Moreover, GRADE was moderate for PHF to prevent wheeze as all included studies with low selection and reporting bias, which showed favorable intervention effects (Supplemental Figure 2, Supplemental Table 4).

#### PHF compared with CMF on allergic outcomes in children aged >2 y

No significant effects of PHF compared with CMF were observed for allergic rhinitis, eczema, asthma, wheeze, and sensitization (Figure 2B).

#### EHF compared with CMF on allergic outcomes in children aged <2 y

**FIGURE 3:**
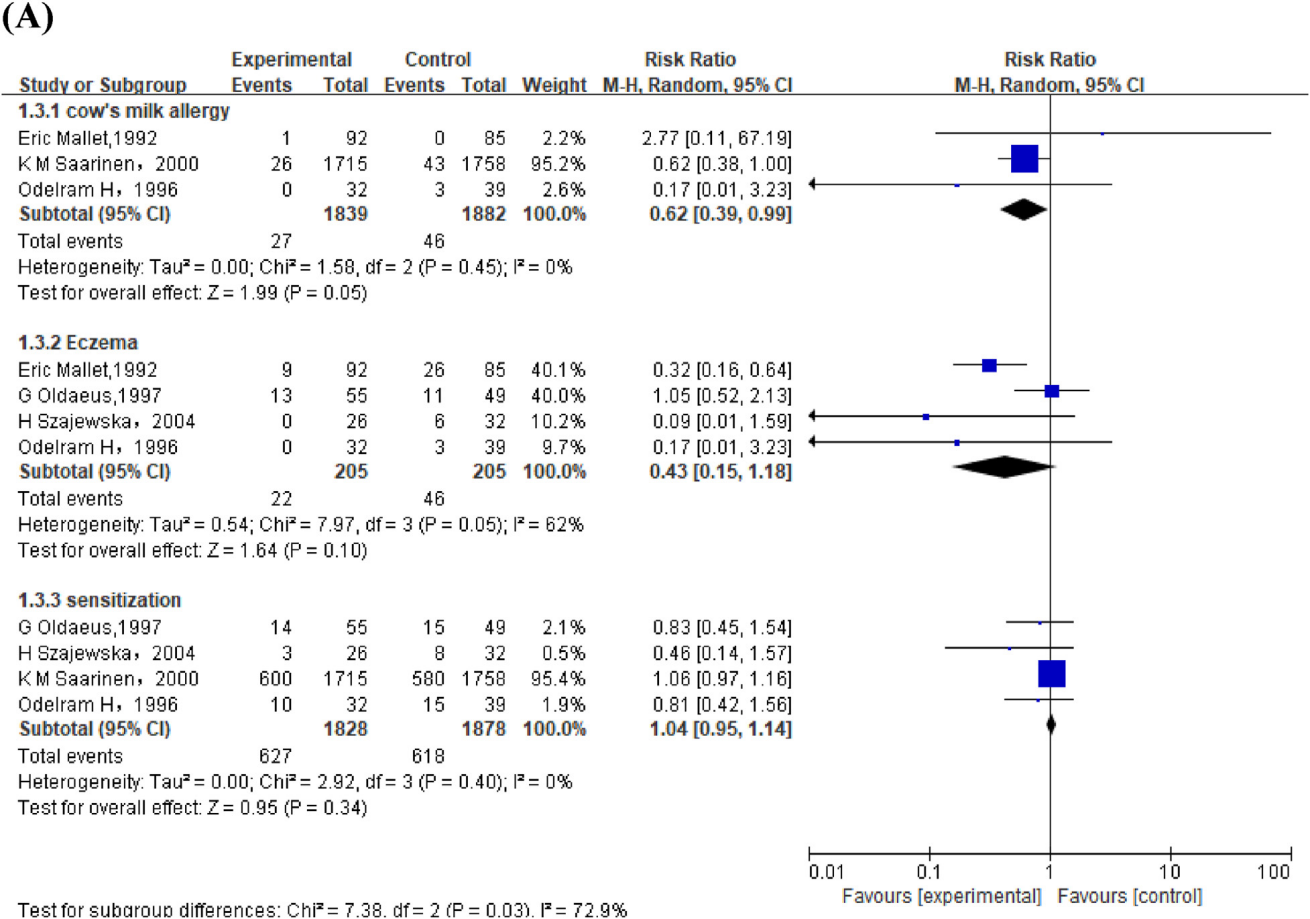

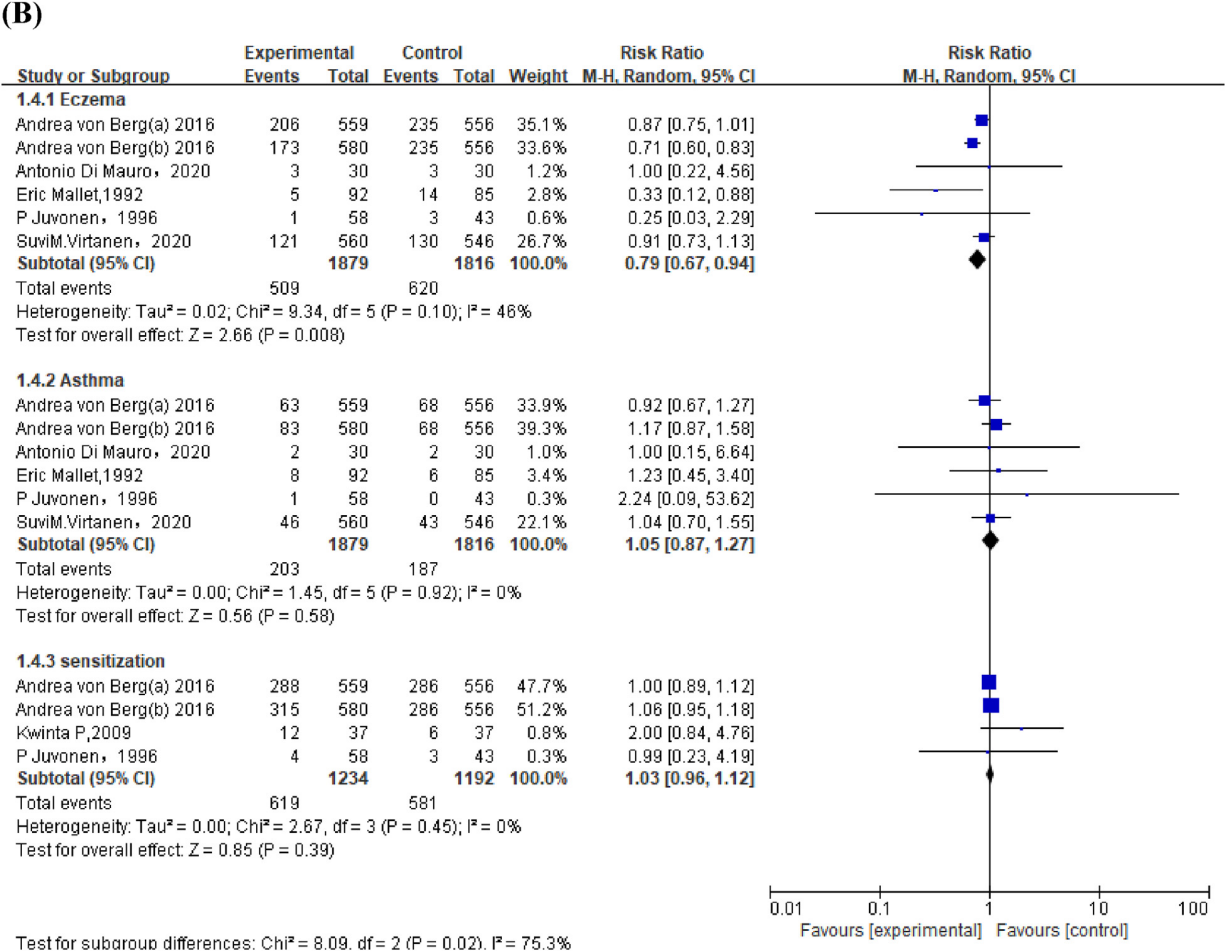
Forest plot of extensively hydrolyzed formula (EHF) compared with cow's milk formula (CMF) on allergic outcomes in (A) children aged <2 y and (B) children aged >2 y. von Berg et al. [25] used both extensively hydrolyzed casein formula [Andrea von Berg (a), 2016] and extensively hydrolyzed whey formula [Andrea von Berg (b), 2016].

Meta-analysis of 3 studies revealed that infants fed EHF had lower risk of cow's milk allergy than those fed CMF in children aged <2 y (RR: 0.62; 95% CI: 0.39, 0.99) (Figure 3A). Because of the high ROB and the inconsistent conclusions among the 3 studies, the GRADE was low (Supplemental Figure 2, Supplemental Table 4).

#### EHF compared with CMF on allergic outcomes in children aged >2 y

Meta-analysis found that compared with CMF, EHF reduced risk of eczema in children, especially in high-risk children aged >2 y (RR: 0.79; 95% CI: 0.67, 0.94 [EHF versus CMF on eczema in children], and RR: 0.75; 95% CI: 0.58, 0.96 [EHF versus CMF on eczema in high-risk children]) (Figure 3B, Supplemental Figure 3). GRADE was moderate for EHF to prevent eczema as most included studies had low or unclear ROB, which demonstrated favorable intervention effects (Supplemental Figure 2, Supplemental Table 4).

### HF feeding compared with breastfeeding

Thirteen studies [26,30,[37], [38], [39],[40], [41], [42], [43], [44],^47^,^48^,^50^] compared the effect of HF compared with that of BM on ADs in children aged <2 y. Of the 58 results focusing on the effect of HF compared with that of BM on total ADs (PHF: 22 and EHF: 36), about half showed a beneficial trend of PHF (48%) or EHF (50%) on total ADs, but none were statistically significant (Supplemental Table 3). The results of the meta-analysis are described further.

#### PHF compared with BM on allergic outcomes in children aged <2 y

**FIGURE 4:**
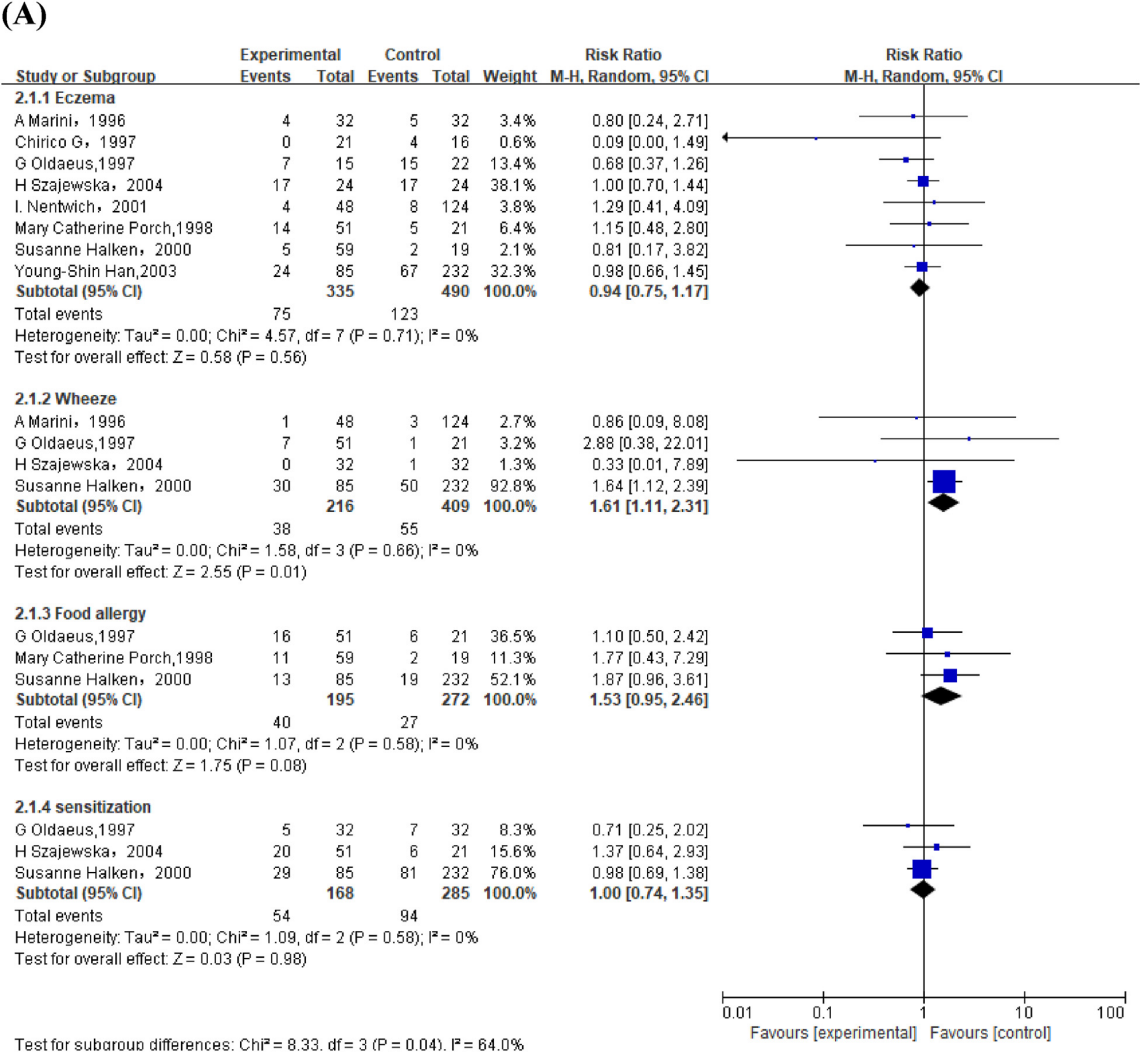

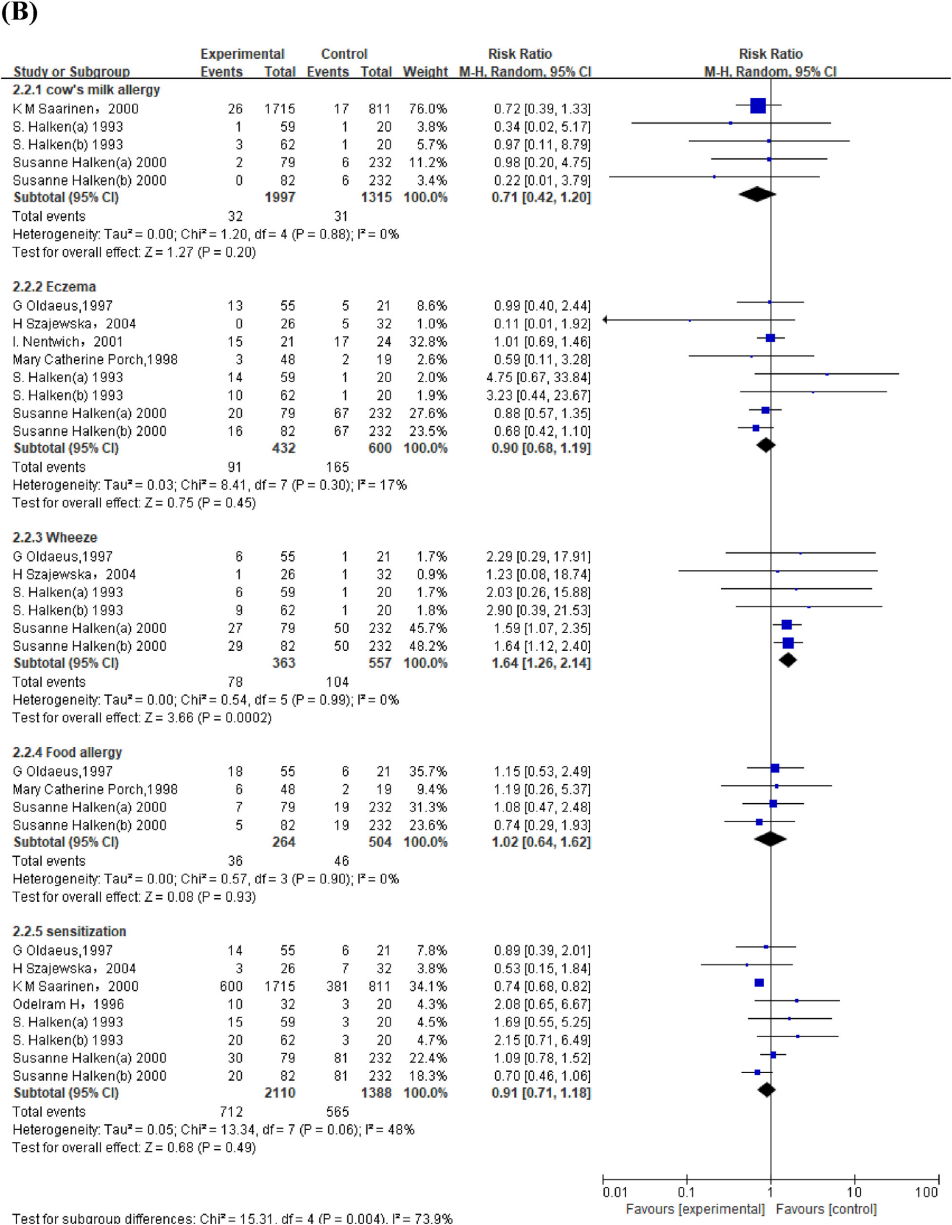
(A) Forest plot of partially hydrolyzed formula (PHF) compared with breast milk (BM) on allergic outcomes in children aged <2 y. (B) Forest plot of extensively hydrolyzed formula (EHF) compared with BM on allergic outcomes in children aged <2 y. ^a^Halken et al. [44] used both extensively hydrolyzed casein formula [S. Halken(a), 1993] and extensively hydrolyzed whey formula [S. Halken (b),1993]. ^b^Halken et al. [50] used both extensively hydrolyzed casein formula [Susanne Halken (a), 2000] and extensively hydrolyzed whey formula [Susanne Halken (b), 2000].

Meta-analysis of 4 studies revealed that PHF increased risk of wheeze (RR: 1.61; 95% CI: 1.11, 2.31) (Figure 4A). GRADE was moderate as 2 of the 4 studies with low selection bias showed the favorable effect of BM (Supplemental Figure 2, Supplemental Table 4).

#### EHF compared with BM on allergic outcomes in children aged <2 y

We identified 4 studies with low performance and reporting bias, which compared the effect of EHF compared with that of BM on wheezing in children aged <2 y. GRADE showed moderate evidence that EHF increased risk of wheeze (RR: 1.64; 95% CI: 1.26, 2.14) (Figure 4B, Supplemental Table 4). Subgroup analysis revealed that neither EHF-W nor EHF-C increased risk of eczema and sensitization (Supplemental Figures 4 and 5).

### Sensitivity analysis and publication bias

Other sensitivity analysis yielded robust results, except for replacing 15-y follow-up article with 3-y follow-up article in the GINI trial. The funnel plot and Begg and Egger tests showed evidence of publication bias for eczema in children aged <2 y (PHF compared with CMF: Begg *P* = 0.124; Egger *P* = 0.013), which was also suggested by trim-and-fill analysis. Trimming did not change the results (Supplemental Figure 6).

## Discussion

This meta-analysis comprehensively compared risk of ADs between children fed different types of infant formulas younger than and older than 2 y. Our results showed low-quality evidence that EHF contributed to lower risk of cow's milk allergy than CMF in children aged <2 y. GRADE indicated moderate evidence that compared with CMF, feeding with PHF reduced risk of eczema in children aged <2 y and EHF decreased risk of eczema after age of 2 y. We also identified moderate systematic evidence indicating that PHF instead of CMF reduced risk of wheeze at age 0–2 y. However, there was low or moderate evidence suggesting that compared with BM, neither PHF nor EHF increased risk of ADs in children aged <2 y, except for wheeze. No significant effects of HF on other ADs were observed in children of any age.

We found that infants consuming EHF in replacement of CMF reduced risk of cow's milk allergy from birth to age 2 y. Previous guidelines [32,52] have recommended using EHF for managing infants with cow milk allergy and preventing allergies. It is biologically plausible that EHF prevents cow's milk allergy. CMF consists of ≥25 different milk proteins, all of which have the potential to act as allergens. Among these, the major allergenic proteins, casein, β-lactoglobulin, and α-lactalbumin contain multiple sensitization epitopes [[53], [54], [55], [56]]. Research [57] has shown that trypsin and chymotrypsin can catalyze the hydrolysis of β-lactoglobulin into smaller peptides and disrupt the linear or spatial structure of allergenic proteins, resulting in a substantial reduction in their allergenic potential. EHF contains peptides with a molecular weight of >95% less than 3000 Da, whereas PHF contains peptides within a molecular weight range of 3000–10,000 Da [58]. Owing to a lower degree of hydrolysis and a higher amount of residual antigenic determinants, PHF may be less effective than EHF in preventing cow's milk allergy [50]. It was worth noting that our study included only 2 studies comparing the effect of PHF with that of CMF on cow's milk allergy, which were insufficient for conducting a meta-analysis. Further research is needed to explore the effectiveness of PHF in preventing cow milk allergy.

Our study found that compared with CMF, early feeding with PHF reduced risk of eczema among children aged <2 y and EHF decreased risk of eczema after age 2 y, which is consistent with previous findings [8,59]. The age-specific differences in the efficacy of PHF and EHF may be attributed to the development of the immune system in children at different stages and the mechanisms of PHF and EHF. From 0 to 2 y, PHF mitigates risk of eczema by modulating the immunologic profile, promoting a balanced T_H_1/T_H_2 cytokine response, and enhancing the development of a robust skin barrier [60,61]. After the age of 2 y, our main analysis did not reveal a significant protective effect of PHF against eczema. However, in sensitivity analysis, when replacing articles from the GINI trial, the results indicated that compared with CMF, PHF reduced risk of eczema in children aged >2 y. It is vital to note that the protective efficacy of PHF against eczema diminished after the age of 2 y, possibly due to age-related enhancement of immune competence and the allergic march [62]. On the contrary, EHF may establish a long-term protective effect on eczema after the age of 2 y through the mechanism of oral immune tolerance, as continuous exposure to smaller peptide segments can further train and modulate the immune system [63]. In children aged 0–2 y, EHF did not have a statistically significant effect against eczema, although the effect size indicated a certain protective trend. It is worth noting that this meta-analysis included only 4 relevant studies with a small sample size. Therefore, caution should be exercised when interpreting this result. We appeal further researchers to investigate the short-term impact of EHF on the prevention of eczema.

Furthermore, subgroup analysis found that compared with CMF, high-risk infants fed PHF or EHF are effective in preventing eczema in children aged younger than or older than 2 y, respectively. Genetic factors play a pivotal role in the development of allergic diseases in offspring [64], and risk of ADs significantly increases when there is a positive family history [65]. In line with our findings, several studies advocated for the utilization of PHF or EHF as a preventive measure against eczema in high-risk infants and children [11]. Nevertheless, among the 10 studies included in our subgroup analysis comparing PHF with CMF, only 1 study involved non–high-risk infants. Therefore, further more robust studies are necessary to confirm the findings on infants from different risk groups.

In this study, to our knowledge, we discovered systematic evidence for the first time that early feeding of PHF instead of CMF prevented wheeze at age 0–2 y. The discrepancies to the previous meta-analysis may stem from differences in the study selection criteria. The Cochrane review [18] included only studies with ≥80% of follow-up, which may have limited power to find the true effects. Another review [16] included trials regardless of the balance of interventions between groups, which may lead to observed results not being the true effect of HF. Several findings support the multiple-hit hypothesis, in which a family history of allergic disease, infant feeding, and other environmental factors could play a key part in the pathogenesis of preschool wheeze [[66], [67], [68]]. Given that infants are not exposed to many environmental toxins and that HF has a low antigenicity, a protective effect of PHF on early childhood wheeze is plausible [42]. However, it should be mentioned that owing to the limited number of studies comparing EHF with CMF, we were unable to draw conclusions regarding the impact of EHF on wheeze. Moreover, in our analysis, all included studies focused on the effect of PHF on wheeze in high-risk infants. Whether HF has similar effect in non–high-risk infants is unknown.

Our study also comprehensively evaluated the effect of PHF or EHF compared with that of BM on ADs. The results showed that neither PHF nor EHF increased risk of ADs in children aged <2 y, except for wheeze. Two interpretations should be taken into consideration. First, BM contains components that interact with the infant’s immune system and intestinal environment, including immunoglobulins, PUFAs, and chemokines [69]. The global consensus [70] strongly advocates exclusive breastfeeding for the first 6 mo of an infant's life, with continued breastfeeding for ≥2 y. Compared with breastfed infants, formula-fed infants had lower bacterial diversity and an altered intestinal microbiota during the initial weeks of life [71]. A previous study also indicted that infants fed CMF instead of BM have higher risk of wheeze in early childhood [72]. Our findings were supported by the national Etude Longitudinale Française depuis l'Enfance birth cohort [73], which showed that compared with BM, HF was associated with higher risk of wheeze but not other ADs. It is worth noting that our findings align with the global consensus on the benefits of breastfeeding, and our research aimed to provide alternative options for infants when breastfeeding is not possible. Second, the progression of ADs can be described by the allergic march [59]. ADs mainly manifest as eczema and wheeze in early childhood and can gradually develop into other allergic symptoms such as allergic rhinitis and asthma with age. Our research is constrained by the length of follow-up of included trails. Thus, we are unable to determine the possible impact of HF compared with that of BM on ADs in children aged >2 y. To answer whether PHF or EHF increases risk of late-onset ADs compared with that of BM, more trial with long-term follow-up is required.

In addition, in our subgroup analysis for meta-analysis, we found that compared with BM, neither EHF-W nor EHF-C increased risk of eczema and sensitization. However, several studies did not provide the protein source of hydrolysate, which limited the subgroup analysis on other ADs. Our findings were supported by previous systematic review [74], which emphasized the benefits of breastfeeding and recommended the use of EHF-C and EHF-W as supplementary feeding for the first 4 mo. Nevertheless, it is worth noting that our findings are specific to the comparison between EHF and BM on eczema and sensitization. The potential preventive effects of EHF-C or EHF-W compared with those of CMF are uncertain and require further investigation. Therefore, our subgroup analyses results have several implications for future research. Future studies comparing PHF or EHF with CMF should provide detailed information on the sources of hydrolyzed proteins. Moreover, previous research elucidated that the preventive efficacy of HF was influenced by multiple factors, including the degree of hydrolysis, the source of hydrolyzed proteins, and the method of hydrolysis [75]. To shine light on the effect of HF, future investigations should strive to encompass not only the degree of hydrolysis but also different hydrolysis methods.

### Strengths and limitations

This systematic review and meta-analysis have several strengths. First, the authors conducted a rigorous screening process to identify relevant articles. Second, only clinical studies were included to minimize confounding. Third, this study is the most comprehensive to compare PHF or EHF with CMF or BM to show the effects of different types of HF on risk of various ADs.

Our study also has several limitations that should be considered. First, only English-language publications were included, which may introduce language bias. However, our included studies covered a broad range of non–English-speaking countries, including those in Asia and Europe. Second, many of the included studies were at an unclear or risk of bias, partly owing to difficulties in blinding participants to the taste differences between HF and CMF. Third, ADs in the included studies were determined using different methods with varying degrees of accuracy and objectivity. However, limited by the number of included articles, subgroup analysis by the methods of outcome assessment was not possible. Finally, the majority of evidence in our analysis received a moderate quality rating. However, certain evidence was categorized as low quality, primarily because of potential biases in study design, inconsistent findings across studies, or imprecise estimates. These findings underscore the imperative for additional research using higher-quality study designs to fortify the evidence. Caution is advised in generalizing the findings of low-quality evidence.

## Conclusion

We found evidence that early feeding EHF in replacement of CMF may reduce risk of cow's milk allergy. Early feeding PHF or EHF may decrease risk of eczema. Moreover, PHF may lessen risk of wheeze in high-risk infants compared with that by CMF but PHF and EHF may increase risk of wheeze but no other ADs compared with that by BM. Given that the majority of studies included high-risk infants, more research on non–high-risk infants is advised before the implementation of this practice.

## Author contributions

The authors’ responsibilities were as follows—LC, IM-YS: were involved in the conception and design of the study, reviewed the manuscript, and assumed primary responsibility for the final content; XXL, JHL: performed the systematic literature search, extracted the data, and quality assessment; XXL: analyzed the data and drafted the manuscript; and all authors: critical revision of the manuscript for important intellectual content and read and agreed to the final version of the manuscript.

## Funding

This work was supported by Huhhot Science & Technology Plan (No. 2021-National Center of Technology innovation for Dairy-4) and supported by National Center of Technology Innovation for Dairy, No. 2022-Ke Yan Gong Guan-4.

## Data availability

All data generated or analyzed during this study are included in this published article and its supplementary information files.

## Conflict of interest

TH, SD, GF, FL, and IM-YS are employed by Inner Mongolia Yili Industrial Group, Yili Maternal and Infant Nutrition Institute (YMINI), Beijing. WY and BL are employed by Inner Mongolia Yili Industrial Group, Hohhot. All other authors report no conflicts of interest.
