# Supplementary material for: Infant Formulas With Partially or Extensively Hydrolyzed Milk Proteins for the Prevention of Allergic Diseases: A Systematic Review and Meta-Analysis of Clinical Trials
Source: Adv Nutr. 2024 Apr 4;15(5):100217. doi: 10.1016/j.advnut.2024.100217 (PMC11063603; doi:10.1016/j.advnut.2024.100217)
Supplement: Multimedia component 1 [file mmc1.docx]

**Infant formulas with partially or extensively hydrolyzed milk proteins for the prevention of allergic diseases: A systematic review and meta-analysis of clinical trials**

**Xiaoxu Li et al. “Online Supplementary Material”**

**Supplementary Figure legends:**

**Supplementary Figure 1:** Forest plot of subgroup analysis based on high-risk versus non-high-risk -- partially hydrolyzed formula versus cow’s milk formula on eczema in children under 2 years of age.

Footnotes: a: Nicolaos Nicolaou,2022 (52) included both high-risk (Nicolaos Nicolaou(a),2022) and non-high-risk (Nicolaos Nicolaou(b),2022) infants.

**Supplementary Figure 2:** Risk of bias summary: review authors' judgements about each risk of bias item for each included study. Each study is represented as an independent row, while different types of bias risks are presented as columns. Each cell in the graph represents the assessment result of each study for a specific bias risk. Each bias risk type has different assessment criteria, typically represented by green, yellow, and red colors indicating low, unclear, and high risk, respectively.

**Supplementary Figure 3:** Forest plot of subgroup analysis based on high-risk versus non-high-risk - extensively hydrolyzed formula versus cow’s milk formula on eczema in children over 2 years of age.

Footnotes: a: Andrea von Berg,2016 (25) used both extensively hydrolyzed casein formula (Andrea von Berg(a),2016) and extensively hydrolyzed whey formula (Andrea von Berg(b),2016).

**Supplementary Figure 4：**Forest plot of subgroup analysis based on casein versus whey dominant hydrolysate - extensively hydrolyzed formula versus breast milk on eczema in children under 2 years of age.

Footnotes: a: S. Halken,1993 (45) used both extensively hydrolyzed casein formula (EHF-C) (S. Halken(a),1993) and extensively hydrolyzed whey formula (EHF-W) (S. Halken(b),1993).

b: Susanne Halken,2000 (51) used both extensively hydrolyzed casein formula (EHF-C) (Susanne. Halken (a),2000) and extensively hydrolyzed whey formula (EHF-W) (Susanne. Halken (b),2000).

**Supplementary Figure 5:** Forest plot of subgroup analysis based on casein versus whey dominant hydrolysate - extensively hydrolyzed formula versus breast milk on sensitization in children under 2 years of age.

Footnotes: a: S. Halken,1993 (45) used both extensively hydrolyzed casein formula (EHF-C) (S. Halken(a),1993) and extensively hydrolyzed whey formula (EHF-W) (S. Halken(b),1993).

b: Susanne Halken,2000 (51) used both extensively hydrolyzed casein formula (EHF-C) (Susanne. Halken (a),2000) and extensively hydrolyzed whey formula (EHF-W) (Susanne. Halken (b),2000).

**Supplementary Figure 6**: The funnel plot can help assess the presence of publication bias. The x-axis represents the effect size estimates (odds ratio). The y-axis represents the standard error. Each data point on the plot corresponds to a study included in the meta-analysis, represented by a dot. The position of the dot reflects the effect size estimate and its precision. The dashed lines represent the symmetry around the overall effect size estimate or the summary effect. The inverted funnel shape illustrates the expected distribution of effect sizes in the absence of publication bias or small-study effects.

**Supplementary Table 1. Search strategy of databases**

**(A)**

| **PubMed：** |
| --- |
| **Infant Formula** |
| ((("Infant Formula"[MeSH Terms] OR "infant formula*"[Title/Abstract] OR "baby formula*"[Title/Abstract] OR "Protein Hydrolysates"[MeSH Terms] OR "protein hydrolysate*"[Title/Abstract] OR "protein hydrolyzate*"[Title/Abstract] OR (("Hydrolysed"[Title/Abstract] OR "Hydrolyzed"[Title/Abstract]) AND ("formula*"[Title/Abstract] OR "protein*"[Title/Abstract] OR "whey"[Title/Abstract] OR "casein"[Title/Abstract])) OR "amino acid formula*"[Title/Abstract] OR "amino acid based formula*"[Title/Abstract] OR "ehf protein human"[Supplementary Concept] OR "PHF"[Title/Abstract]) |
| **Allergic diseases** |
| ("asthma"[Title/Abstract] OR "wheeze"[Title/Abstract] OR "intestinal hypersensitivity"[Title/Abstract] OR "atopy"[Title/Abstract] OR "atopic dermatitis"[Title/Abstract] OR "eczema"[Title/Abstract] OR "Anaphylaxis"[MeSH Terms] OR "anaphylactoid reaction*"[Title/Abstract] OR "Allergy"[Title/Abstract] OR "Allergies"[Title/Abstract] OR "allergic urticaria"[Title/Abstract] OR "allergic rhinitis"[Title/Abstract] OR "Hypersensitivity"[MeSH Terms] OR "Hypersensitivities"[Title/Abstract] OR "sensitization"[Title/Abstract]) |
| **Children** |
| ("girl"[Title/Abstract] OR "boy"[Title/Abstract] OR "offspring"[Title/Abstract] OR "neonate*"[Title/Abstract] OR "newborn*"[Title/Abstract] OR "Preschool"[Title/Abstract] OR "Infant"[MeSH Terms] OR "infant*"[Title/Abstract] OR "Infancy"[Title/Abstract] OR "Children"[Title/Abstract] OR "Child"[MeSH Terms] OR "early life"[Title/Abstract])) |
| **Excluded animal experiments** |
| ("animals"[MeSH Terms] NOT "humans"[MeSH Terms]) |

**(B)**

| **EMBASE：** |
| --- |
| **Infant Formula** |
| ('artificial milk'/exp OR 'infant formula*’: ab,ti OR 'baby formula*':ab,ti OR 'protein hydrolysate'/exp OR ((hydrolysed:ab,ti OR hydrolyzed:ab,ti) AND ('formula'/exp OR 'protein'/exp OR 'whey'/exp OR 'casein'/exp)) OR 'amino acid-based formula*':ab,ti OR 'amino acid formula*':ab,ti OR ehf:ab,ti OR phf:ab,ti OR aaf:ab,ti) |
| **Allergic diseases** |
| ('eczema'/exp OR 'wheezing'/exp OR 'asthma'/exp OR 'atopic dermatitis'/exp OR 'allergic rhinitis'/exp OR 'allergic urticaria'/exp OR 'hypersensitivity'/exp OR hypersensitivities:ab,ti OR 'allergy'/exp OR allergies:ab,ti OR 'anaphylaxis'/exp OR 'anaphylactoid reaction*':ab,ti OR 'sensitization'/exp OR 'atopy'/exp) |
| **Children** |
| ('boy'/exp OR 'girl'/exp OR offspring:ab,ti OR neonate*:ab,ti OR newborn*:ab,ti OR 'newborn'/exp OR preschool*:ab,ti OR 'early life':ab,ti OR 'infant'/exp OR 'infant*':ab,ti OR 'child'/exp OR children:ab,ti OR 'infancy'/exp OR 'baby'/exp) |
| **Excluded animal experiments** |
| ('animal'/exp not 'human'/exp) |

**(C)**

| **Cochrane:** |
| --- |
| **Infant Formula** |
| ((Infant Formula):ti,ab,kw OR (baby formula):ti,ab,kw OR (Protein Hydrolysate*):ti,ab,kw OR (Protein Hydrolyzate*):ti,ab,kw OR (amino acid formula*):ti,ab,kw OR (EHF):ti,ab,kw OR (PHF):ti,ab,kw OR (AAF):ti,ab,kw OR (((Hydrolysed):ti,ab,kw OR (Hydrolyzed):ti,ab,kw) AND ((formula*):ti,ab,kw OR (Protein*):ti,ab,kw OR [mh whey] OR [mh caseins])) |
| **Allergic diseases** |
| ([mh Asthma] OR (Wheezing):ti,ab,kw OR (intestinal hypersensitivity):ti,ab,kw OR (atopy):ti,ab,kw OR (atopic dermatitis):ti,ab,kw OR [mh eczema] OR [mh Anaphylaxis] OR (anaphylactoid reaction):ti,ab,kw OR (anaphylactoid shock):ti,ab,kw OR (Allergy):ti,ab,kw OR (allergic urticaria):ti,ab,kw OR (allergic rhinitis):ti,ab,kw OR [mh Hypersensitivity] OR (sensitization):ti,ab,kw) |
| **Children** |
| ([mh child] OR (children): ti,ab,kw OR (infant*):ti,ab,kw OR (infancy):ti,ab,kw OR (offspring):ti,ab,kw OR (earil life):ti,ab,kw OR (Newborn*):ti,ab,kw OR (neonate*):ti,ab,kw OR (preschool):ti,ab,kw OR (boy*):ti,ab,kw OR (girl*):ti,ab,kw) |

**(D)**

| **WOS:** |
| --- |
| **Infant Formula** |
| ("Infant Formula*" OR "baby formula*" OR "Protein Hydrolysate*" OR "Protein Hydrolyzate*" OR "Protein Hydrolysate*" OR ((Hydrolysed OR Hydrolyzed) AND (formula* OR Protein* OR whey OR casein)) OR "amino acid formula*" OR "amino acid-based formula*" OR EHF OR PHF) |
| **Allergic diseases** |
| (asthma OR wheeze OR "intestinal hypersensitivity” OR atopy OR "atopic dermatitis" OR eczema OR Anaphylaxis OR "anaphylactoid reaction*" OR Allergy OR Allergies OR "allergic urticaria" OR "allergic rhinitis" OR Hypersensitivity OR Hypersensitivities OR sensitization) |
| **Children** |
| (girls OR boys OR offspring OR Neonate* OR Newborn* OR Preschool* OR Infant* OR Infancy OR Children OR Child OR "early life”) |
| **Excluded animal experiments** |
| NOT (animal not human) |

**Supplementary Table 2. The details of the articles from the same trail.**

| **ID**  **(trail)** | **Article** | ***NO.（EG/CG）** | **Age at**  **outcome** | **outcomes** | **Main findings** |
| --- | --- | --- | --- | --- | --- |
| **4** | Y.Vandenplas,  1988 [29] | PHF-W:28  CMF:30 | 6-month-old | IgE levels,  allergic symptoms | Mean IgE levels on day 5 (0.8-1.2 U/ml) were similar in all groups. IgE levels increased less significantly during the first months of life in the infants on HF compared with the groups with CMF (*P* < 0.01) or breast-feeding (*P* < 0.05). |
|  | Y.Vandenplas,  1995* [28] | PHF-W:28  CMF:30 | 6-month-old  1-year-old;  3-year-old;  5-year-old | allergic rhinitis,  Wheeze,  Eczema,  sensitization | ①At 6 months, the prevalence of cow's milk protein (CMP) sensitivity was significantly decreased in the hydrolysate group (7% versus 43%; *P*: 0.002). At the age of 12 (21% versus 53%; *P*: 0.029), 36 (25% versus 57%; *P*: 0.018) and 60 months (29% versus 60%; *P*: 0.016) there was still a significant difference in the number of atopic manifestations, if calculated cumulatively.  ②Eczema was less frequent in the whey-hydrolysate group, but only during the I st year of life, suggesting a decreased prevalence of CMF sensitivity.  ③The results of this study support the hypothesis that allergy prevention is antigen specific |
| **7** | A.von Berg,  2003 [21] | PHF-W:557  EHF-W:559  EHF-C:580  CMF:556 | 1-year-old | Eczema,  sensitization | ①The incidence of allergic manifestation was significantly reduced by using EHF-C compared with CMF (9% vs 16%; adjusted OR, 0.51; 95% CI, 0.28-0.92).  ②the incidence of AD was significantly reduced by using EHF-C (OR, 0.42; 95% CI, 0.22-0.79) and partially hydrolyzed whey formula (OR, 0.56; 95% CI, 0.32-0.99). |
|  | Andrea von Berg,  2006 [22] | PHF-W:461  EHF-W:494  EHF-C:485  CMF:486 | 3-year-old | Asthma,  Eczema,  Sensitization | ①A signiﬁcant reduction of the incidence of eczema was achieved with the extensively hydrolyzed casein formula(odds ratio [95% CI], 0.67 [0.45-0.99], and with the partially hydrolyzed whey formula(odds ratio, 0.76 [0.52-1.11]).  ②None of the formulas reduced the incidence of asthma. |
|  | Andrea von Berg,  2010 [23] | PHF-W:459  EHF-W:455  EHF-C:455  CMF:455 | 6-year-old | Asthma,  Rhinitis,  Eczema,  Sensitization | ①In the intent-to-treat analysis the relative risk of sensitization compared with CMF was 0.82 (95% CI, 0.70-0.96) for partially hydrolyzed whey formula, 0.90 (95% CI, 0.78-1.04) for extensively hydrolyzed whey formula, and 0.80 (95% CI, 0.69-0.93) for extensively hydrolyzed casein formula.  ②The corresponding ﬁgures for atopic eczema were 0.79 (95% CI, 0.64-0.97), 0.92 (95% CI, 0.76-1.11), and 0.71 (95% CI, 0.58-0.88), respectively. |
|  | Andrea von Berg,  2013 [24] | PHF-W:366  EHF-W:362  EHF-C:377  CMF:346 | 10-year-old | Asthma,  Rhinitis,  Eczema,  Sensitization | ①The relative risk for the cumulative incidence of sensitization in the intention-to-treat analysis was 0.87 (95% CI, 0.77-0.99) for PHF-W, 0.94 (95% CI, 0.83-1.07) for EHF-W, and 0.83 (95% CI, 0.72-0.95) for EHF-C compared with cow’s milk formula.  ②The corresponding ﬁgures for atopic eczema/dermatitis were 0.82 (95% CI, 0.68-1.00), 0.91 (95% CI, 0.76-1.10), and 0.72 (95% CI, 0.58-0.88), respectively |
|  | Andrea von Berg,  2016* [25] | PHF-W:352  EHF-W:347  EHF-C:346  CMF:332 | 15-year-old | Asthma,  Rhinitis,  Eczema,  Sensitization,  Food allergy | ①Between 11 and 15 years, the prevalence of asthma was reduced in the EHF-C group compared to CMF (OR 0.49, CI 0.26–0.89).  ②The cumulative incidence of AR was lower in EHF-C (RR 0.77, 95% CI 0.59–0.99) and the AR prevalence in PHF-W (OR 0.67, 95% CI 0.47–0.95) and EHF-C (OR 0.59,95% CI 0.41–0.84).  ③The cumulative incidence of eczema was reduced in PHF-W (RR 0.75, 95% CI 0.59–0.96) and EHF-C (RR 0.60, 95% CI 0.46–0.77). |
| **18** | K M Saarinen,  1999 [31] | EHF-W:1737  CMF:1789  BM:824 | 2-month-old  6-7-month-old | Cow’s milk allergy | The cumulative incidence of CMA in the infants’ fed CMF was 2.4% compared with 1.7% in the pasteurized human milk group (odds ratio [OR], 0.70; 95% confidence interval [CI], 0.44-1.12) and 1.5% in the whey hydrolysate group (OR, 0.61; 95% CI, 0.38-1.00). |
|  | K M Saarinen,  2000* [30] | EHF-W:1737  CMF:1789  BM:824 | 2-month-old  6-7-month-old | Cow’s milk allergy,  sensitization  TGF-β1  IgE | ①The cumulative incidence of CMA, verified by a CM elimination-challenge test, was 2.4% in the CMF, 1.7% in the pasteurized human milk and 1.5% in the whey hydrolysate group.  ②Colostrum TGF- β1 may inhibit IgE and cell mediated reactions and promote IgG-IgA antibody production to CM in infants prone to developing CMA. |
| **19** | P Juvonen，  1996* [26] | EHF-C:58  CMF:43  BM:43 | 3-year-old | Cow’s milk allergy,  Eczema;  Asthma;  Sensitization; | No differences were found in allergy (cow’s milk allergy, eczema, asthma) between the three groups. |
|  | P Juvonen，  1994 [27] | EHF-C:58  CMF:43  BM:43 | 24 month-age | Serum IgG， IgE antibodies  and allergic symptoms | ①The levels of IgG antibodies to b-lactoglobulin (IgG-BLG) and bovine serum albumin (IgG-BSA) were higher in the CMF and the HM groups than in the CHF group for up to two years.  ②The cumulative incidence of allergic symptoms was 17% (21/129) and the prevalence was 13% (16/129) at two years of age. |

a: abbreviations: BM, Breast milk; CG, Control group; CI, Confidence interval; CMA, Cow’s milk allergy; CMF, Cow’s milk formula; EG, Experimental group; EHF, Extensively hydrolyzed formula; PHF-W, Partially hydrolyzed whey formula; EHF-C, Extensively hydrolyzed casein formula; EHF-W, Extensively hydrolyzed whey formula; HF, Hydrolyzed cow's milk formula; PHF, Partially hydrolyzed formula; RR, Risk ratio.

b: * indicated this article was selected for meta-analysis.

**Supplementary Table 3.** **Percentage (%) of included studies demonstrating the beneficial effect of HF versus CMF or BM on allergic diseases.**

| **Comparison** | **Type of allergic outcomes: number of results** | | | | | | | | **% with statistically significant and favorable effect** | | | | | | | | **% with favorable effect (P<0.05 not necessary)** | | | | | | | |
| --- | --- | --- | --- | --- | --- | --- | --- | --- | --- | --- | --- | --- | --- | --- | --- | --- | --- | --- | --- | --- | --- | --- | --- | --- |
|  | **C** | **R** | **E** | **A** | **W** | **FA** | **S** | **T** | **C** | **R** | **E** | **A** | **W** | **FA** | **S** | **T** | **C** | **R** | **E** | **A** | **W** | **FA** | **S** | **T** |
| **HF VS CMF (≤2 years)** | | | | | | | | | | | | | | | | | | | | | | | | |
| HF | 4 | 2 | 13 | 3 | 8 | 3 | 9 | 44 | 25 | 0 | 15 | 0 | 13 | 0 | 11 | 11 | 75 | 50 | 77 | 100 | 88 | 0 | 67 | 70 |
| PHF | 2 | 2 | 10 | 1 | 6 | 2 | 5 | 28 | 0 | 0 | 20 | 0 | 0 | 0 | 20 | 11 | 100 | 50 | 80 | 100 | 100 | 0 | 60 | 71 |
| PHF-W | 2 | 2 | 5 | 0 | 2 | 1 | 3 | 15 | 0 | 0 | 20 | - | 0 | 0 | 33 | 7 | 0 | 50 | 80 | - | 100 | 0 | 33 | 67 |
| EHF | 3 | 0 | 4 | 2 | 2 | 1 | 4 | 16 | 33 | - | 25 | 0 | 50 | 0 | 0 | 13 | 67 | - | 75 | 100 | 50 | 0 | 75 | 69 |
| EHF-C | 1 | 0 | 1 | 1 | 0 | 0 | 0 | 3 | 0 | - | 100 | 0 | - | - | - | 33 | 0 | - | 100 | 100 | - | - | - | 67 |
| EHF-W | 2 | 0 | 1 | 0 | 0 | 0 | 2 | 5 | 50 | - | 0 | - | - | - | 0 | 0 | 100 | - | 100 | - | - | - | 50 | 80 |
| **HF VS CMF (＞2 years)** | | | | | | | | | | | | | | | | | | | | | | | | |
| HF | 1 | 7 | 12 | 9 | 4 | 4 | 7 | 44 | 0 | 14 | 33 | 11 | 0 | 0 | 0 | 11 | 0 | 57 | 92 | 44 | 75 | 0 | 71 | 66 |
| PHF | 0 | 4 | 6 | 3 | 3 | 1 | 3 | 20 | - | 0 | 33 | 33 | 0 | 0 | 0 | 10 | - | 75 | 83 | 67 | 100 | 0 | 100 | 85 |
| PHF-W | 0 | 3 | 4 | 3 | 1 | 1 | 3 | 15 | - | 0 | 25 | 33 | 0 | 0 | 0 | 13 | - | 67 | 50 | 67 | 100 | 0 | 100 | 80 |
| EHF | 1 | 3 | 6 | 6 | 1 | 3 | 4 | 24 | 0 | 33 | 33 | 0 | 0 | 0 | 0 | 13 | 0 | 33 | 83 | 33 | 0 | 0 | 25 | 50 |
| EHF-C | 1 | 2 | 4 | 4 | 1 | 1 | 3 | 16 | 0 | 0 | 25 | 0 | 0 | 0 | 0 | 18 | 0 | 0 | 100 | 0 | 0 | 0 | 50 | 41 |
| EHF-W | 0 | 1 | 1 | 1 | 0 | 1 | 1 | 5 | - | 100 | 100 | 0 | - | 0 | 0 | 0 | - | 100 | 100 | 100 | - | 0 | 0 | 40 |
| **HF VS BM (≤2 years)** | | | | | | | | | | | | | | | | | | | | | | | | |
| HF | 6 | 3 | 16 | 5 | 10 | 7 | 11 | 58 | 0 | 0 | 0 | 0 | 0 | 0 | 0 | 0 | 0 | 33 | 63 | 40 | 10 | 14 | 55 | 48 |
| PHF | 1 | 1 | 8 | 2 | 4 | 3 | 3 | 22 | 0 | 0 | 0 | 0 | 0 | 0 | 0 | 0 | 0 | 0 | 63 | 50 | 50 | 0 | 67 | 45 |
| PHF-W | 1 | 1 | 3 | 1 | 1 | 2 | 1 | 11 | 0 | 0 | 0 | 0 | 0 | 0 | 0 | 0 | 0 | 0 | 33 | 50 | 100 | 0 | 100 | 36 |
| EHF | 5 | 2 | 8 | 3 | 6 | 4 | 8 | 36 | 0 | 0 | 0 | 0 | 0 | 0 | 0 | 0 | 0 | 50 | 63 | 33 | 0 | 25 | 50 | 50 |
| EHF-C | 2 | 1 | 3 | 1 | 2 | 2 | 2 | 14 | 0 | 0 | 0 | 0 | 0 | 0 | 0 | 0 | 0 | 0 | 100 | 0 | 0 | 0 | 50 | 29 |
| EHF-W | 3 | 1 | 2 | 1 | 2 | 1 | 4 | 15 | 0 | 0 | 0 | 0 | 0 | 0 | 0 | 0 | 0 | 100 | 50 | 100 | 0 | 100 | 50 | 40 |

a: abbreviations: C, cow’s milk allergy; R, rhinitis; E, eczema; A, asthma; W, wheeze; FA, food allergy; S, sensitization; T, total; HF, hydrolyzed cow's milk formula; PHF, partially hydrolyzed

formula; EHF, extensively hydrolyzed formula; PHF-W, partially hydrolyzed whey formulas; EHF-C, extensively hydrolyzed casein formula; EHF-W, extensively hydrolyzed whey formula.

b: - indicates no results.

| **Comparison** | **Quality of the evidence (GRADE)** | | | | | | |
| --- | --- | --- | --- | --- | --- | --- | --- |
|  | **Cow’s milk allergy** | **Rhinitis** | **Eczema** | **Asthma** | **Wheeze** | **Food Allergy** | **Sensitization** |
| **HF VS CMF (≤2 years)** | | | | | | | |
| PHF | -- | -- | moderate^1^ | -- | moderate^1^ | -- | moderate^1^ |
| EHF | low^1,2^ | **--** | low^1,3^ | -- | -- | **--** | low^1,3^ |
| **HF VS CMF (＞2 years)** | | | | | | | |
| PHF | -- | moderate^1^ | moderate^1^ | low^1,2^ | moderate^1^ | -- | moderate^1^ |
| EHF | -- | -- | moderate^1^ | moderate^1^ | -- | -- | low^1,2^ |
| **HF VS BM (≤2 years)** | | | | | | | |
| PHF | -- | -- | low^1,2^ | -- | low^1,2^ | moderate^1^ | low^1,2^ |
| EHF | moderate^1^ | -- | low^1,2^ | -- | moderate^1^ | moderate^1^ | low^1,2^ |

**Supplementary Table 4. Quality of the evidence (GRADE)**

a: GRADE Working Group grades of evidence, **High quality:** Further research is very unlikely to change our confidence in the estimate of effect. **Moderate quality:** Further research is likely to have an important impact on our confidence in the estimate of effect and may change the estimate. **Low quality:** Further research is very likely to have an important impact on our confidence in the estimate of effect and is likely to change the estimate. **Very low quality:** We are very uncertain about the estimate.

b: ^1^ This meta-analysis includes studies that suffer from unclear or high bias, ^2^ more than 20% of studies have inconsistent conclusions, ^3^ more than 20% of studies have a wide CI, -- indicates that there are too few studies to conduct meta-analysis.


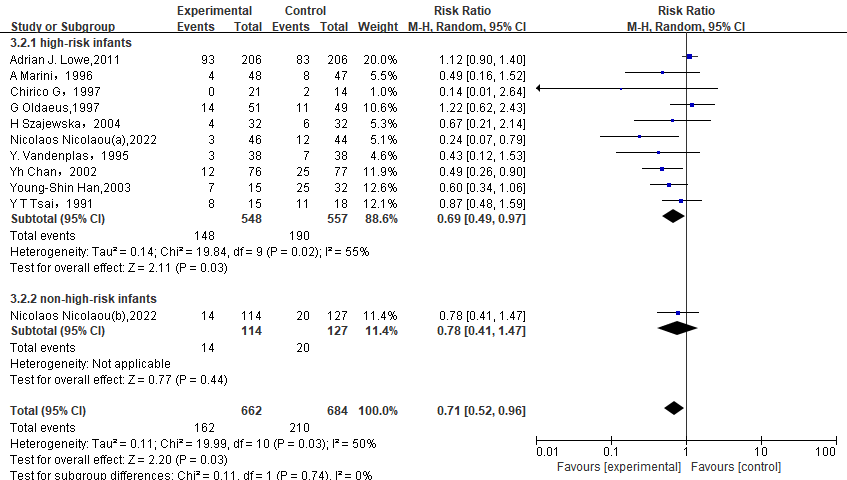


## Supplementary Figure 1. Forest plot of subgroup analysis based on high-risk versus non-high-risk infants - partially hydrolyzed formula versus cow’s milk formula on eczema in children under 2 years of age.

**
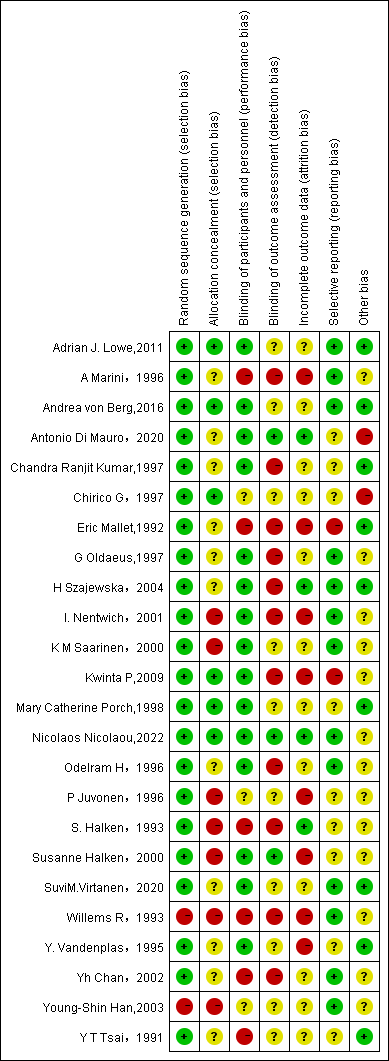
**

**Supplementary Figure 2. Risk of bias.**


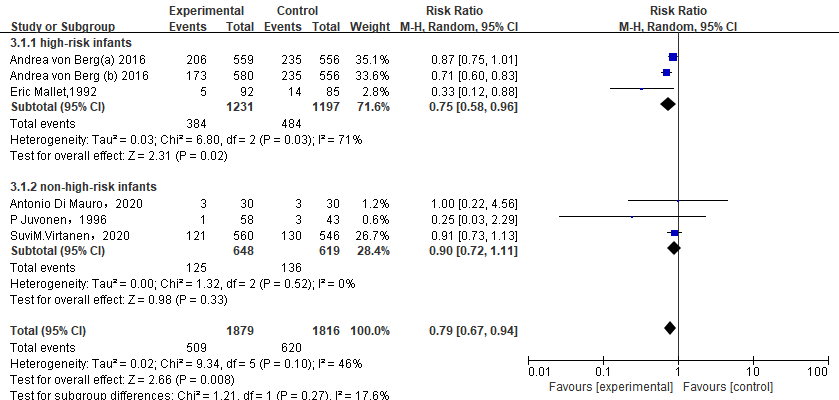


## Supplementary Figure 3. Forest plot of subgroup analysis based on high-risk versus non-high-risk - extensively hydrolyzed formula versus cow’s milk formula on eczema in children over 2 years of age.


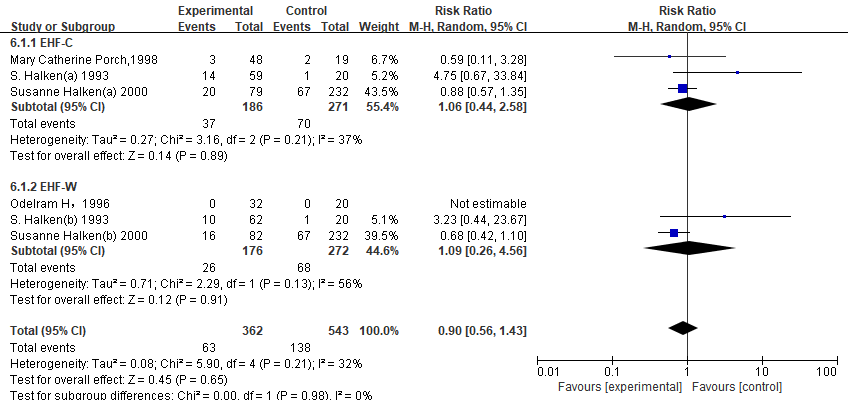


**Supplementary Figure 4. Forest plot of subgroup analysis based on casein versus whey dominant hydrolysate - extensively hydrolyzed formula versus breast milk (BM) on eczema in children under 2 years of age.**


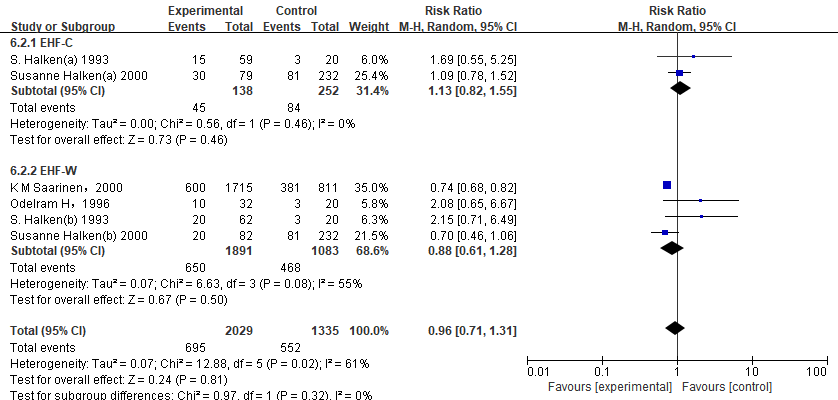


**Supplementary Figure 5. Forest plot of subgroup analysis based on casein versus whey dominant hydrolysate - extensively hydrolyzed formula versus breast milk (BM) on sensitization in children under 2 years of age.**

**Supplementary Figure 6. Funnel plot of comparison partially hydrolyzed formula versus cow's milk formula on eczema in children under 2 years of age.**
